# The Enigmatic PE/PPE Multigene Family of Mycobacteria and Tuberculosis Vaccination

**DOI:** 10.1128/IAI.00969-16

**Published:** 2017-05-23

**Authors:** Michael J. Brennan

**Affiliations:** Aeras, Rockville, Maryland, USA; University of Florida

**Keywords:** tuberculosis, PE/PPE family of genes, vaccines

## Abstract

The genome of Mycobacterium tuberculosis, the bacterium responsible for the disease tuberculosis, contains an unusual family of abundant antigens (PE/PPEs). To date, certain members of this multigene family occur only in mycobacteria that cause disease. It is possible that the numerous proteins encoded by these mycobacterial genes dictate the immune pathogenesis of this bacterial pathogen. There is also evidence that some of these antigens are present at the cell surface and that they affect the pathology and immunology of the organism in many ways. Also, they elicit both antibodies and T cells, they may be involved in antigenic variation, and they may be good candidates for vaccines and drugs. However, since they are plentiful and extremely homologous, these PE/PPEs are very challenging to study, and it is difficult to be certain what role(s) they have in the pathogenesis of tuberculosis. Consequently, how to develop treatments like vaccines using these antigens as candidates is complex.

**If I could keep death by tuberculosis away by building a building**

**I would have built that building a long time ago**

—Bheki, a tuberculosis patient (and carpenter) in South Africa

## GENERAL FEATURES OF PE/PPEs

In 1998, the Mycobacterium tuberculosis genome highlighted, for the first time, the presence of genes grouped into two large families that were shown to comprise approximately 7% of the genome size (1, 2). This was a surprise to the field of mycobacteriology and led to the speculation that this multitude of repetitive genes, found mostly in slow-growing pathogenic mycobacteria, likely influence the function and immunopathogenicity of M. tuberculosis. Based on the presence of conserved Pro-Glu (PE) and Pro-Pro-Glu (PPE) motifs at the N termini of the proteins, the genes encoding these proteins were named *pe* and *ppe*, respectively. The laboratory strain of M. tuberculosis H37Rv contains 99 *pe* genes, 61 of which are in the PE-PGRS (polymorphic GC-rich sequence) subfamily (3, 4), a subfamily earlier used for fingerprinting M. tuberculosis strains) and 69 *ppe* genes. Studies have shown that this number can vary for different strains of M. tuberculosis. The corresponding proteins are further classified into subfamilies, often depending on the amino acid sequence at the C terminus (2, 5).

The proteins belonging to the PE family share a highly conserved N-terminal domain about 90 to 110 amino acids in length. The PE family is further divided into the PE and PE_PGRS subfamilies (2, 5). PE-PGRS proteins are characterized by the presence of a polymorphic domain, rich in Gly-Gly-Ala/Gly-Gly-Asn amino acid repeats, which can vary in sequence and size. *pe* and PGRS genes are found scattered throughout the genome and are mostly not cotranscribed with other genes. Conversely, many of the *pe* genes are adjacent to *ppe* genes, and a number of studies have demonstrated that these *pe-ppe* couplets are coexpressed (6). At least some of the corresponding proteins are found as heterodimers that are present on the cell surface or secreted (7, 8).

**FIG 1:**
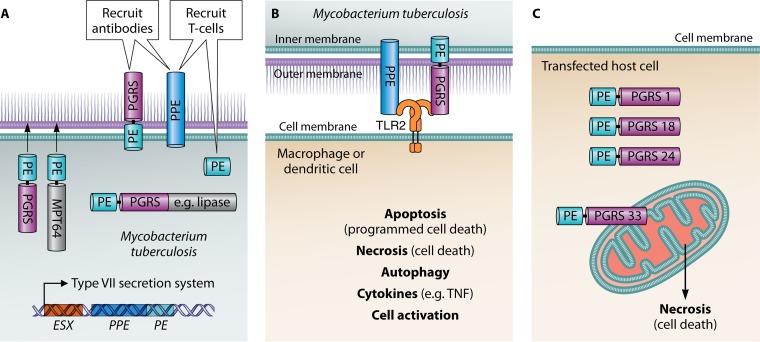
Schematic showing known PE/PPE bacterial ligands and how they may interact with mycobacterial or host receptors. (A) PE-PGRS and PPEs are found in the outer membrane matrix of mycobacteria, and they can elicit cross-reactive antibodies. The PPEs and PEs can elicit T cells. PEs can act like chaperones directing PGRS and other heterologous proteins like MPT64 to the outer membrane of mycobacteria. Some PE-PGRSs also have a domain at the C terminus, such as a lipase, which has enzymatic activity. PPEs and PEs often interact as “couplets” and are found associated with ESX domains in mycobacterial genes. The type VII secretion system is known to secrete PE/PPEs as well as other antigens. (B) PE-PGRS and PPEs interact with TLR2 receptor on macrophages and dendritic cells. This binding has been shown to cause apoptosis, necrosis, autophagy, release of cytokines like tumor necrosis factor alpha (TNF-α) and cell activation. (C) Necrosis of the cells resulting from the interaction of PE-PGRS 33 with mitochondria. Other PE-PGRSs like PE-PGRS 1, 18, and 24 do not interact with mitochondria.

The PPE proteins are also characterized by the presence of a highly conserved N-terminal domain that is approximately 180 amino acids in length, and similar to the PE domain, seems to play a key role in driving protein localization or secretion (9, 10). Many PPE proteins appear to be coexpressed with the PE partner (as couplets, as mentioned) and belong to the PE/PPE pair subfamily; others are encoded by genes found scattered in the chromosome. Downstream of the conserved N-terminal PPE domain, many of the PPEs contain a major polymorphic tandem repeat (MPTR) region characterized by multiple C-terminal repeats of the amino acid sequence motif Asn-X-Gly-X-Gly-Asn-X-Gly (11). Other subfamilies of PPEs exist, such as those containing an SVP motif (2) The C termini of PE/PPEs may also contain other sequences, as is observed with lipases or regions with other enzymatic functions (9, 12) (Fig. 1).

## EVOLUTION OF PE/PPEs

There is evidence that homologous recombination between genetic sequences emerged by duplication events, and this may have shaped the evolution of *pe* and *ppe* genes in M. tuberculosis (11). There is also good evidence that they have evolved along with the ESX regions of M. tuberculosis in studies done by Gey van Pittius and coworkers (11) (Fig. 1A). There are five multigene regions (type VII secretion systems) that contain PEs and PPEs and that are involved in the secretion of proteins like ESAT-6 (M. tuberculosis early secreted antigenic target of 6 kDa), an M. tuberculosis virulence factor often used as a vaccine antigen (8, 13, 14). Publications indicate that the ESX family arose from a plasmid precursor in fast-growing mycobacteria, and they contain virulence factors that can interact with the macrophage (15, 16). The type VII secretion system can also translocate a number of proteins, including PE/PPEs across the impermeable outer membrane matrix of slow-growing mycobacteria (13). Additionally, it is known that the ESX1 domain (RD1, the region of difference 1) is lacking in all Mycobacterium bovis BCG strains and contributes to the attenuation of BCG (17). Expansion of the PE/PPE families may have provided the genes needed for adaptation of M. tuberculosis to the human host (18, 19, 20). Remarkably, almost nothing is known about host cell receptors for PE/PPE ligands with the exception of interactions with Toll-like receptor 2 (TLR2), which is discussed below.

## FUNCTION, LOCATION, AND IMMUNOGENICITY OF PE/PPEs

Over the past decade, evidence has accumulated from a number of investigators on the function, location, immunogenicity, and evolution of PE/PPEs. The reader is directed to excellent PE/PPE discussions in the publications (2, 11, 21) and the chapters by Delogu et al. (22) and Brennan et al. (23) for a discussion on the other aspects of the PE/PPEs. Some of the early studies indicated that certain PE/PPEs were found at the cell surface and in a position to interact with other bacteria and members of the host immune system (24, 25, 26) (Fig. 1). Other studies demonstrated that domains, especially at the C terminus, were mutated, and this could result in deletions that were surprisingly, still in-frame and produced viable proteins (27).

An unexpected number of PE/PPEs have been shown to interact with TLR2, and these can have immune-modulating properties and promote macrophage activation (Fig. 1B). Both PPEs and PE-PGRSs have been shown to bind to TLR2 and to activate both macrophages and dendritic cells and induce the release of cytokines that promote apoptosis and necrosis of host cells (28, 29, 30). These studies with TLR2 suggest that PE/PPEs could play an important role in TB pathogenesis and provide immunity needed for a vaccine. Recent data, obtained with PE_PGRS33, indicate that even a small PGRS region, containing a few GGA-GGN repeats, can activate the TLR2-dependent entry into macrophages (31). It is worth noting that mycobacteria such as Mycobacterium canettii, which lack certain PE/PPEs, can also signal through TLR2 (32, 33).

Interestingly, it was demonstrated that a single PE_PGRS could be mutated and result in a specific nonredundant phenotype, even though the proteins are often 50% homologous or more (25). This suggests that knockouts in PE/PPEs may be informative. In addition, the PEs have been shown to act like a chaperone, carrying heterologous (Mpt64) or homologous (PE-PGRS) polypeptides to the surface of the bacteria (34, 35). Furthermore, certain PE/PPEs have been shown to aggregate near the poles of mycobacteria similar to ESX1, and this could be related to a specific function in M. tuberculosis (36, 37). Another PE-PGRS, PE-PGRS 30, has also been shown to arrest macrophage acidification and growth of M. tuberculosis within macrophages (38). It is also possible that one PE/PPE antigen may make a good tuberculosis (TB) vaccine, for example, PE-PGRS 62 has been shown to have a role in replication and persistence of the bacillus, and is highly conserved, which may make it a good vaccine candidate (20).

It has been known for some time that PE/PPEs can be localized to the surface of bacteria (5), and recent evidence suggests that the stability and integrity of the capsule found outside mycobacteria are due in part to the ESX5 type VII secretion system and to PPE 10 (39). This relationship between PE/PPEs and the type VII secretion systems as well as the ESX domains appears to be very important to mycobacteria particularly during evolution (as mentioned) and to the role of PE/PPEs as immunogens.

It has been demonstrated that expression of PE/PPEs is controlled by a number of transcriptional regulators such as multiple SIGs, PhoP and DevRS as outlined by Fishbein and coworkers (18) and Voskuil et al. (40). Variable transcription can also contribute to PE/PPE expression as observed for example with the inverse expression of PE-PGR 16 and 26 following infection (41). All of these systems as well as the expression of highly redundant homologous proteins can influence PE/PPE immunogen manifestation and delineate their use in vaccines. The inclination for PE/PPEs to diverge due to single nucleotide polymorphisms (SNPs), deletions, and insertions (27) can also augment their use in vaccines. The increased tendency of PE/PPE genes to recombine is an important observation, as these genes may be able to change rapidly in response to selective pressure.

The role of the PE/PPE family in antigenic variation of M. tuberculosis has been postulated since their discovery, but this hypothesis is controversial (18, 20, 42, 43). More studies on PE/PPE need to be performed before we understand the difference between polymorphism diversity within clinical isolates compared to antigenic variation in the organism caused by this multigene family.

Cadieux et al. (44) showed that PE-PGRS 33 colocalizes to the mitochondria of transfected cells, a phenomenon dependent on the linker region and the PGRS domain, but not the PE domain. Using different genetic fusions and chimeras, it was also demonstrated that a direct correlation exists between localization to the host mitochondria and the induction of cell death. Interestingly, other PE-PGRS proteins tested, including PE-PGRS 1, 18, and 24, did not bind to the mitochondria of transfected cells (Fig. 1C). Considering the importance of primary necrosis and dissemination during natural infection of M. tuberculosis, the PE-PGRS 33 protein may play a crucial role in the pathogenesis of tuberculosis. Also, disruption of the PE_PGRS47 (Rv2741) gene led to attenuated growth of M. tuberculosis *in vitro* and *in vivo*, and the PE_PGRS47 mutant showed enhanced major histocompatibility complex (MHC) class II-restricted antigen presentation (45). Deletion of PE_PGRS47 implicated this protein in the inhibition of autophagy in infected-host phagocytes. Therefore, besides having a role in apoptosis and necrosis, PE/PPEs may also participate in autophagy (Fig. 1B).

## STAGE GATES

Aeras in the United States and the TuBerculosis Vaccine Initiative in Europe and others are using stage gates to choose and evaluate TB vaccines (46). Certain criteria are used to decide which vaccine candidates progress into efficacy and safety trials in humans. One of the most difficult areas during early discovery stages is showing that a candidate provides adequate immunogenicity and efficacy, particularly in preclinical models. For the PE/PPEs, it is difficult to determine which antigens should be included, if they should be combined, or if they should be developed and administered as a live vaccine or as a subunit vaccine together with an adjuvant or be viral vectored or be given as a DNA vaccine.

## CLINICAL STUDIES OF TB VACCINES CONTAINING PPEs

**FIG 2:**
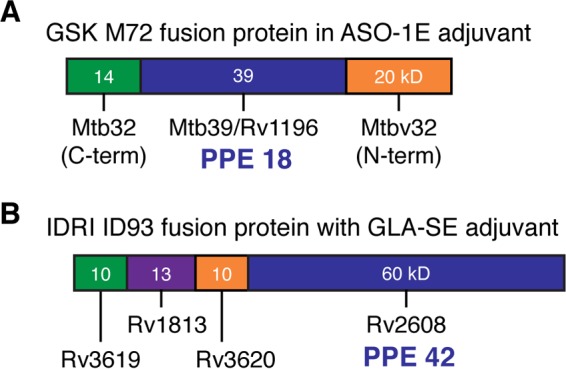
Schematic showing the position of PPE 18 (Mtb39a) in the M72 GSK vaccine (A) or PPE 42 (Rv2608) as part of the IDRI ID93 vaccine (B) which are presently in clinical trials.

Unlike certain vaccines already in the clinic, a vaccine composed of PE/PPE antigens may be valuable, even though our understanding of the specific functions or immune responses to these antigens is limited. Vaccines composed in part of PPE genes are already in phase II studies (Fig. 2). The M72 vaccine sponsored by GSK (47) and originally developed by Corixa (48) contains PPE 18 also known as Mtb39a (Rv1196; Fig. 2). The M. tuberculosis genome has two additional highly homologous PPE genes, Mtb39b and Mtb39c, as well as many other PPEs which contain homologous regions which could cross-react. Studies of recombinant genetic changes occurring in clinical isolates of PPE 18 protein are found in regions of PPE 18 reported to be potential T-cell epitopes (49). This suggests that the Mtb72F vaccine may not recognize a certain proportion of M. tuberculosis strains present in the natural population.

Also, the ID93 vaccine sponsored by IDRI, contains a PPE, PPE 42 (Rv2608; Fig. 2) (50). Evidence thus far indicates that the PPE genes in both vaccines are immune dominant likely because they cross-react with many PPE genes throughout the M. tuberculosis genome through homologous regions in the PPEs. Cross-reactive immunity among this multigene family could be an important attribute contributing to an effective TB vaccine (51). It will be important to study these vaccines carefully, as recent evidence indicates that variability in the PPE protein in GSK's M72 vaccine, even at the N terminus, is common among circulating clinical M. tuberculosis strains (52). The impact of these PPE antigens in the particular TB vaccines remains to be determined, but clinical data should be available in the near future.

A composite of PE and PPE proteins may be effective as a vaccine, but that remains to be determined. Strong et al. (6) showed that PE 25/PPE 41 present in ESX1 needs to associate with each other to be functional, and this may affect immunogenicity as well. Similarly, PE/PPE couplets found in other ESX regions present in M. tuberculosis may also be a sensible choice for TB vaccines. There may be cross-reactivity among proteins in the family, as already demonstrated for PPEs in clinical vaccines, and this could be important. In addition, ESX5 is important in many pathogenic mycobacteria, and the PE/PPE found in this region may also be influential, as discussed below (13) (Fig. 1A).

## THE LIVE *M. TUBERCULOSIS* ΔPPE/PE25-PE19 MUTANT VACCINE

The live M. tuberculosis vaccine lacking PE/PPEs was developed by the laboratories at the University of Pisa and Institute Pasteur (53), and described in detail by Sayes et al. (54) deserves further discussion because five PE/PPEs, located in the ESX5 region, are specifically removed. MTBVAC (55), the first live M. tuberculosis vaccine, has already been studied in phase 1 trials, and it has been shown to be safe (56). This M. tuberculosis vaccine, however, is a regulatory mutant, and several virulence factors may be lacking in the final strain. In the M. tuberculosis ΔPPE/PE25-PE19 mutant, three PPEs (PPE 25, 26, and 27) and two PEs (PE 18 and 19) are specifically removed in the laboratory strain H37Rv. Before it can be used as a vaccine, a second mutation will also need to be introduced for safety as suggested by regulatory agencies (57). The nature of this second mutation is unknown at this time, but it will be very important. Insertion of a regulatory mutation, like that in MTBVAC, could result in the variable expression of numerous antigens and difficulties in characterizing the final product. SCID mouse data have demonstrated that M. tuberculosis ΔPPE/PE25-PE19 is attenuated, and surprisingly, mouse efficacy studies have indicated that it is more protective than M. bovis BCG (54). Attenuation is likely due to the five PPE/PEs that have been removed, but it is more difficult to understand why the M. tuberculosis ΔPPE/PE25-PE19 mutant is more protective than the parent. As indicated by Sayes et al. (51), this is probably due to cross-reactive immunity, because the vaccine recognizes a number of PPE/PEs, including those that are missing in the mutant vaccine. Alternatively, if the function of the PPE/PEs was to assist M. tuberculosis in evading host immunity, the lack of PE/PPEs could also result in improved efficacy. However, protection is likely due to the induction of specific CD4 T cells against PE/PPE antigens that are cross-reactive with other nonshared PE/PPEs and that are secreted or placed on the cell surface by the ESX-5 type VII secretion system (13). Documentation of the history of the strain and additional preclinical studies, including protection in the guinea pig and maybe in a nonhuman primate (NHP) need to be completed before this new live M. tuberculosis vaccine based on PE and PPE antigens can enter clinical trials.

## FUTURE DIRECTIONS

There is good evidence that PEs contain epitopes for a TH1 response which makes them logical candidates for a TB vaccine (Fig. 1). Human T cell epitopes appear to be concentrated in the conserved PE domain which exhibits little sequence diversity in phylogenetically distinct isolates of M. tuberculosis (58). Some members of the PE/PPE family can also specifically elicit CD8 T cells as shown by a proteomic peptide library (59). There is also a distinctive study of a DNA vaccine consisting of only a PE gene that is protective in the mouse TB model (60). Therefore, PEs may be good candidates for a TB vaccine.

It is plausible that both T cell and antibody responses are needed to control intracellular as well as extracellular stages of M. tuberculosis in tuberculosis. Since PPEs and PE-PGRSs elicit cross-reactive antibodies against the repeating domains of these proteins, these antigens could be added to a PE TB vaccine that induces T cells to make a more effective vaccine. Also, PE-PGRSs have been shown to promote entry of M. tuberculosis into macrophages via TLR2, an important step in the pathogenesis of tuberculosis (61). PE-PGRS and other PE/PPEs can be constitutively expressed in M. tuberculosis, and the linker region (the GRPLI motif that links the N-terminal PE with the PGRS domain) is also found in all PE-PGRSs. For these reasons, a TB vaccine may benefit from the addition of PE-PGRSs.

Vaccine efficacy can be reduced if nonvaccine strains become prevalent (62). Some believe that the popular use of BCG vaccine, for example, is responsible for the emergence of the seven circulating strains of M. tuberculosis that most frequently cause TB. This is not likely to happen with effective vaccines composed of protective PE/PPE antigens because they are so numerous and are likely to be cross-reactive. The clinical trials of M72 and ID93, in the near future, should inform us if PPE-containing TB vaccines are effective and if the immune responses against PPE are valuable. Meanwhile, further study of vaccines like M. tuberculosis ΔPPE/PE25-PE19 and TB vaccines containing other PE/PPE antigen cassettes should inform the field. It will be important to establish whether PE and PPEs should be part of an effective TB vaccine candidate and also how best to select the new TB vaccine candidate if there is no correlate.
